# Proteomic analysis of leaves and roots during drought stress and recovery in *Setaria italica* L.

**DOI:** 10.3389/fpls.2023.1240164

**Published:** 2023-10-11

**Authors:** Hui Gao, Weina Ge, Lin Bai, Ting Zhang, Ling Zhao, Jingshi Li, Jiangjie Shen, Ningwei Xu, Haoshan Zhang, Genping Wang, Xiaohu Lin

**Affiliations:** ^1^ Hebei Key Laboratory of Crop Stress Biology, Department of Life Science and Technology, College of Marine Resources and Environment, Hebei Normal University of Science and Technology, Qinhuangdao, Hebei, China; ^2^ Institute of Millet Crops, Hebei Academy of Agriculture and Forestry Sciences/Key Laboratory of Genetic Improvement and Utilization for Featured Coarse Cereals(Co-construction by Ministry and Province), Ministry of Agriculture and Rural Affairs/Key Laboratory of Minor Cereal Crops of Hebei Province, Shijiazhuang, China; ^3^ College of Life Sciences, North China University of Science and Technology, Tangshan, China; ^4^ State Key Laboratory of Genetic Engineering and Collaborative Innovation Center for Genetics and Development, School of Life Sciences, Institute of Biomedical Sciences, Fudan University, Shanghai, China; ^5^ College of Landscape and Tourism, Hebei Agricultural University, Baoding, China

**Keywords:** drought stress, leaf, root, proteomic, *Setaria italica* L

## Abstract

Drought is a major environmental factor that limits agricultural crop productivity and threatens food security. Foxtail millet is a model crop with excellent abiotic stress tolerance and is consequently an important subject for obtaining a better understanding of the molecular mechanisms underlying plant responses to drought and recovery. Here the physiological and proteomic responses of foxtail millet (cultivar Yugu1) leaves and roots to drought treatments and recovery were evaluated. Drought-treated foxtail millet exhibited increased relative electrolyte leakage and decreased relative water content and chlorophyll content compared to control and rewatering plants. A global analysis of protein profiles was evaluated for drought-treated and recovery treatment leaves and roots. We also identified differentially abundant proteins in drought and recovery groups, enabling comparisons between leaf and root tissue responses to the conditions. The principal component analysis suggested a clear distinction between leaf and root proteomes for the drought-treated and recovery treatment plants. Gene Ontology enrichment and co-expression analyses indicated that the biological responses of leaves differed from those in roots after drought and drought recovery. These results provide new insights and data resources to investigate the molecular basis of tissue-specific functional responses of foxtail millet during drought and recovery, thereby significantly informing crop breeding.

## Introduction

Climate change has gradually brought about a hotter and more arid world, with an increasing occurrence of abiotic stresses on organisms (FAO, 2019). In particular, drought is one of the most serious environmental threats and limits crop growth, thereby threatening food security (Parry et al., 2005; Du et al., 2022; Martínez-Goñi et al., 2022). Consequently, it is urgently needed to better understand the mechanisms of crop responses to drought stress in order to improve crop drought tolerance (Lobell et al., 2008; Zhao & Running, 2010).

Foxtail millet (*Setaria italica* L.) is one of the most suitable crops for dryland agriculture, and genomic data for several cultivars are now available (Lee et al., 2007). Additionally, foxtail millet is a C4 plant that has a higher photosynthetic efficiency than C3 plants under high temperature and drought conditions and also serves as an ideal model for C4 plant research due to its small genome, short reproductive cycle, and accessible genetic transformation tools (Bennetzen et al., 2012; Yang et al., 2020). Furthermore, foxtail millet is a model plant to investigate the molecular mechanisms of drought tolerance because it exhibits greater drought tolerance and water use efficiency than other crops such as wheat, maize, and sorghum (Pan et al., 2018).

Previous studies have investigated the morphological and physiological changes of foxtail millet in response to drought (Tang et al., 2017; Pan et al., 2018; Zhang et al., 2022). Several genome-wide transcriptomic studies have also elucidated the genetic basis of drought tolerance in foxtail millet (Qi et al., 2013). However, the proteomic responses of foxtail millet to drought stress remain largely unexplored despite the identification of numerous drought-responsive genes and noncoding RNAs at the transcriptional level (Qi et al., 2013; Tang et al., 2017; Pan et al., 2018; Zhang et al., 2022). Proteomic approaches have been recently applied to detect global changes of proteins due to drought stress in many crops, thereby revealing the molecular mechanisms of drought tolerance, such as in rice (Salekdeh et al., 2002; Ali & Komatsu, 2006), maize (Trevisan et al., 2015), barley (Sugimoto & Takeda, 2009; Kausar et al., 2012), and sunflower (Rauf, 2008; Ghaffari et al., 2013). Moreover, a previous comparative proteomics study identified 321 proteins that were responsive to drought stress in whole foxtail millet seedlings (Pan et al., 2018). These proteins were involved in various physiological and metabolic processes, including in stress and defense responses, photosynthesis, and carbon metabolism.

Plant leaves and roots sense and respond to drought differently. Roots are the first to detect drought signals, as they directly interact with soils, and then trigger various drought responses, such as root developmental and structural changes and stomatal movements in aerial plant organs (Gupta et al., 2020). Therefore, it is necessary to distinguish between aboveground and underground components to investigate the drought tolerance mechanisms of foxtail millet. Several recent studies have focused on the mechanisms of plant leaf and root responses to drought stress—for example, the drought-responsive genes of Dongxiang wild rice were identified by constructing leaf and root cDNA libraries (Deng et al., 2018). Furthermore, oligonucleotide microarrays were used to investigate the transcriptomes of chickpea seedling leaves and roots under drought stress (Wang et al., 2012). In addition, high-throughput RNA sequencing (RNA-seq analysis) was utilized to assess the transcriptomic changes in the leaves and roots of *Prunus persica* under drought stress (Ksouri et al., 2016). Transcriptional responses to drought stress were likewise investigated in the leaves and roots of drought-sensitive lentil (*Lens culinaris*) using comparative RNA-seq analysis (Morgil et al., 2019). These studies have demonstrated significant differences in the responses of leaves and roots to drought stress among different plants. Moreover, the increased instability of rainfall patterns has necessitated the investigation of crops under drought and rewatering conditions to help stabilize dry farming yields (Tatar et al., 2015). Rewatering effects on plant development have been investigated in various crops, including maize (Chen et al., 2016), barley (Sicher et al., 2012), and rice (Zhou et al., 2007).

Previous studies have indicated that foxtail millet roots and leaves respond differently to drought, but few studies have evaluated leaf and root responses to drought stress and rewatering at the proteome level. Here the hypothesis that the protein abundance patterns of leaves and roots in foxtail millet differed in response to drought and rewatering was evaluated. Specifically, proteomic differences between foxtail millet leaves and roots were compared under drought stress and recovery conditions. Differentially abundant proteins in leaves and roots were significantly distinct and were associated with diverse stress pathways in both drought and recovery conditions. These insights and results provide a useful framework for better understanding the molecular basis of tissue-specific roles in drought and the recovery responses of plants.

## Materials and methods

### Plant materials and growth conditions

The foxtail millet cultivar Yugu1 was bred at the Anyang Academy of Agricultural Sciences (Henan, China) and acquired from Prof. Xianmin Diao (Chinese Academy of Agricultural Science). The genome sequence of Yugu1 was previously generated and made publicly available (Bennetzen et al., 2012). Mature non-dormant Yugu1 seeds were germinated in petri dishes covered with moist filter papers for 36 h and then transplanted into polyvinyl chloride pots (8 cm × 8 cm × 10 cm in height) with 300 g of soils comprising nutrient soils and loamy sand mixed in a 1:1 ratio (v/v). The plants were grown under long-day greenhouse conditions (16 h light at 28°C and 8 h dark at 24°C), with a photon flux density of 350–700 μmol/m^2^/s (Yang et al., 2020). In total, 400 uniformly developed seedlings with six leaves were selected for the drought stress and recovery treatment experiments. The control groups contained drought stress (control-D) and recovery (control-R) controls that were watered daily to ensure soil moisture, with approximately 40% to 50% soil volumetric water content (Tang et al., 2017). The plants were initially grown in the drought stress (drought) and recovery (recovery) groups by withholding water until the soil gravimetric water content reached 20% of field capacity (lasting for 8 days). After drought treatment, seedlings from the control-D and drought groups were immediately harvested, followed by the separation of leaves and roots and subsequent freezing in liquid nitrogen and storage at −80°C. The seedlings of the recovery group after drought treatment were rewatered for 24 h and harvested when the soil moisture was restored to 40%–50%. The seedlings of the control-R group plants were also harvested at the same time. The leaves and roots of seedlings from the control-R and recovery groups were then immediately separated and frozen in liquid nitrogen, followed by storage at −80°C, respectively. All samples were randomly collected from four independent experiments. A soil moisture analyzer instrument (TDR300; Spectrum, Aurora, IL, USA) was used to measure the soil water content.

### Physiological measurements

The top second leaves from different plants in the same treatments were used for physiological measurements, including relative water content (RWC), relative electrolyte leakage (REL), and chlorophyll content. Each sample group consisted of four biological replicates. The RWC, REL, and chlorophyll content were measured as previously described (Harborne, 1988; Cao et al., 2007; Mukami et al., 2019). Automatic measurement of total root morphology indicators, including root length and surface area, was conducted with the WinRHIZO Reg 2009c software program (Regent Instruments Inc., Quebec, Canada) as previously described (Xiao et al., 2020).

### Protein extraction, trypsin digestion, and LC–MS/MS analysis

Leaves and roots from the control-D, drought, control-R, and recovery groups were collected and ground to fine, smooth powder in liquid nitrogen and then continuously ground after adding the extraction buffer that included 20 mM KCl (AiYan, product no. AY42565-500g), 20 mM HEPES (pH 7.4; Sigma, product no. H4034-100G), 1 M hexylene glycol (MedChemExpress, product no. HY-B0903), 0.5% (v/v) Triton X-100 (Invitrogen, product no. HFH10), 1% (v/v) thiodiglycol (DR. EHRENSTORFER, product no. CAS CDCT-GA09010352ME), 50 μM spermine (GLPBIO, product no. GC14953-5g), 125 μM spermidine (Coolaber, product no. CS10431-1g), 1 mM PMSF (Thermo Scientific, code no. 36978), and a protease inhibitor cocktail (Roche; product no. 4693116001) (Tang et al., 2020). Homogenates were filtered through a double-layered Miracloth. The flow-through from filtering was centrifuged at 15,000×*g* for 10 min, followed by collection of the supernatant as whole-tissue extract. Protein concentrations were determined with Bradford protein assays (Sangon Biotech, product no. C503031). Extracts from each sample (100 μg of protein) were reduced with 10 mM dithiothreitol at 56°C for 30 min and alkylated with 10 mM iodoacetamide at room temperature in the dark for an additional 30 min. The samples were digested with trypsin using a filter-aided sample preparation method (Wis'niewski et al., 2009). Tryptic peptides were separated using a homemade reverse-phase C18 column. The peptides were eluted, vacuum-dried (Concentrator Plus, Eppendorf), and analyzed by liquid chromatography–tandem MS (LC–MS/MS).

The protein samples were analyzed on an Orbitrap Fusion Lumos instrument (Thermo Fisher Scientific, Rockford, IL, USA) coupled with high-performance liquid chromatography (EASY-nLC 1200 System Instrument, Thermo Fisher Scientific). Dried peptide samples were then redissolved in solvent A (0.1% formic acid in water) and loaded onto a trap column (100 μm × 2 cm, particle size of 3 μm; pore size, of 120 A°C; SunChrom, USA) with a maximum pressure of 280 bar. The samples were then separated on a 150 μm × 12 cm silica microcolumn (particle size, of 1.9 μm; pore size of 120 A°C; SunChrom, USA) with a gradient of 5%–35% mobile phase B (acetonitrile and 0.1% formic acid) and a flow rate of 600 nL/min for 75 min. MS analysis was conducted in data-dependent mode using the full-scan mode (300–1,400 m/z) and acquired using an Orbitrap mass analyzer at a mass resolution of 12,000 at 200 m/z. The automatic gain control target was set to 3e6 and followed by up to 20 data-dependent MS/MS scans with higher-energy collision dissociation (target of 5e3 ions, maximum injection time of 20 ms, isolation window of 1.6 m/z, and a normalized collision energy of 27%). Data were acquired using the Xcalibur software program version 4.2.28.14 (Thermo Fischer Scientific).

### Peptide identification and protein quantification

Raw peptide files were processed using Maxquant (Cox & Mann, 2008), and the data were searched against the *Setaria italica* V2.2 genome from the Joint Genome Institute protein database (https://phytozome.jgi.doe.gov/pz/portal.html), allowing mass tolerances of 20 ppm for precursors and 0.5 Da for product ions, with up to two missed cleavages allowed. A label-free, intensity-based absolute quantification (iBAQ) method was used to generate label-free protein quantifications. The normalized abundance of a specific protein across samples was identified as the percentage of the total (FOT). FOTs were calculated by dividing a protein’s iBAQ by the total iBAQ of all identified proteins in a sample. The FOT values were multiplied by 10e5 for ease of presentation (Zhang C et al., 2017). Proteins comprising at least one unique peptide and two high-confidence peptides (mascot ion score >20) in a minimum of two biological replicates in one treatment group and a peptide-level false discovery rate (FDR) of 1% were chosen for further investigation.

### Bioinformatics and statistical analyses

The analysis in this study focused on the proteins identified in >2 replicates of samples. Owing to the missing protein in each sample being different, we then filled the missing value by the 1/10 of the minimum value of the whole data matrix. Correlation analysis was conducted with the corrplot package (version 0.84) for R. Principal component analysis (PCA) was conducted using the ClustVis tool (https://biit.cs.ut.ee/clustvis/). GO analysis was also conducted for sets of enriched genes using the TBtools package (Chen et al., 2020). Consensus clustering was implemented by Consensus Cluster Plus (version 1.38.0) for R. The schematics of metabolic pathways and the proteomics data were generated using the MapMan program (Thimm et al., 2004).

### RNA isolation and quantitative real-time PCR

Total RNA was extracted from foxtail millet samples using an RNAiso kit according to the manufacturer’s protocols (TaKaRa, code no. 9108), followed by generation of first-strand cDNAs by reverse transcription with First Strand cDNA Synthesis Kit (TaKaRa, product no. 6210A). Real-time PCR assays were conducted using FastStart Universal SYBR Green Master mix (Roche, product no. 4913914001) on a 7500 real-time PCR instrument (Applied Biosystems). To reduce background noise, ROX reference dye was added to the FastStart Universal SYBR Green Master mix. Each PCR reaction contained 10 μL of SYBR Green Master mix, 1 μL (5 μM) of gene-specific primers, and diluted cDNA, with a total volume of 20 μL. The *SitEF-1a-2* gene was used as an internal reference, as previously described (Tang et al., 2017). All primer annealing temperatures were 56°C, and the primers are shown in **Supplementary Table S1**. Each PCR assay was conducted with three technical replicates. The delta–delta Ct method was used to calculate the relative gene expression (Livak & Schmittgen, 2001).

## Results

### The physiological responses of Yugu1 to drought stress and recovery conditions

To determine the effects of drought treatment, the morphological and physiological characteristics of Yugu1 seedlings were analyzed. Healthy and stably growing Yugu1 seedlings were divided into control and drought groups. The seedlings were cultivated under normal conditions in the control group (control-D, soil moisture of approximately 46.9%). In the drought group, the seedlings were subjected to progressively increased soil water depletion until the soil gravimetric water level was below 20% on the 8th day (**Supplementary Figures S1A, B**). Leaf rolling, a clear visible sign of drought stress, was observed in Yugu1 seedlings (drought condition, soil moisture of about 18.7%) (**Supplementary Figure S1A**). The Yugu1 seedlings were rewatered on the 8th day after withholding water, leading to unrolling of leaves within 1 day after rewatering (recovery condition, soil moisture of around 45.4%), while plants grew healthily under control conditions (control-R, soil moisture of about 48.3%) (**Supplementary Figures S1A, B**). The physiological analysis of Yugu1 seedlings under drought stress showed a decrease in RWC and chlorophyll content and an increase in REL (**Supplementary Figures S1C–E**).

The root morphologies of Yugu1 seedlings were also evaluated (**Supplementary Figure S2A**). Under drought stress, the total root lengths of Yugu1 significantly increased compared with the control group. The root lengths of the recovery group plants were slightly longer than those of the control-R group, although no significant statistical differences were observed (**Supplementary Figure S2B**). Root surface areas, as determined by root lengths and diameters, were significantly higher in drought treatment plants compared to those of the control-D group (**Supplementary Figure S2C**). In addition, the root surface areas of control-R and recovery group plants progressively increased with root system growth, with no statistically significant differences between the two groups (**Supplementary Figure S2C**). Thus, drought stress caused leaf damage in plants, including reduced RWC and chlorophyll content, along with increased REL, but rewatering significantly reversed these effects. The root morphologies also significantly changed under drought stress based on root lengths and surface areas. Proteomic analysis was consequently conducted using the leaves and roots of Yugu1 seedlings under drought and recovery conditions.

### A region-resolved reference map of Yugu1 seedlings

To generate a Yugu1 proteome reference under drought and recovery conditions, Yugu1 leaves and roots were collected after drought and recovery. Four biological replicates for proteomic analyses were collected for the treatments, including control-D, drought, control-R, and recovery in aboveground (leaf) and underground (root) organs of foxtail millet.

**Figure 1:**
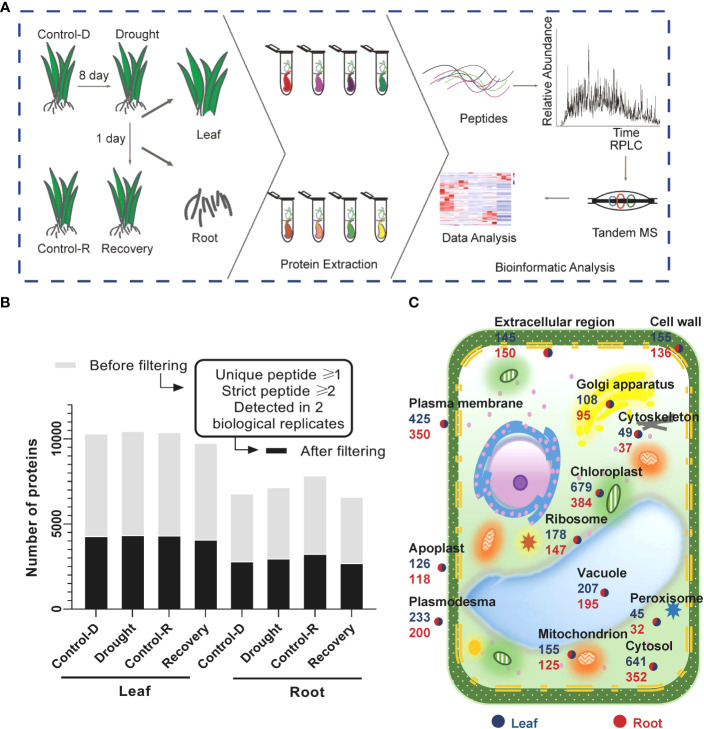
brief summary of the proteomic analysis of drought stress and drought recovery in foxtail millet (*Setaria italic* L.) **(A)** Illustration of the sample collection, preparation, and LC–MS/MS. Foxtail millet tissue samples were collected from aerial (leaves) and underground (root) parts, respectively. Different tissues were in-solution digested with trypsin, and the resulting peptides were separated for LC–MS/MS analysis. **(B)** Proteomic datasets filtered for at least one unique peptide and two strict peptides in a minimum of two biological replicates in one treatment group. The light gray color displays the number of gene products (GPs) before filtering; the dark gray color shows the number of GPs after filtering. **(C)** Subcellular distribution of gene products annotated with Gene Ontology in leaf and root tissues.

A high-resolution quadrupole Orbitrap mass spectrometer (Orbitrap Fusion Lumos) was used to conduct LC–tandem MS (LC–MS/MS) to comprehensively map the Yugu1 proteome (**Figure 1A**, **Supplementary Table S2**). The four biological replicates for the same tissues and treatments were comparable, as revealed by high interexperiment correlation coefficients (**Supplementary Figure S3**). A total of 5,982 and 4,171 gene products (GPs) were identified in leaf and root tissues, respectively. GPs that contained at least one unique peptide and two high-confidence peptides and were present in at least two biological replicates in each treatment group were identified, resulting in about 4,261 GPs in leaf tissues and 2,934 in root tissues with high confidence (**Figure 1B**). The subcellular localization and abundances of GPs were specifically analyzed in leaf and root tissues (**Figure 1C**). Many proteins belonged to extracellular areas or apoplasts, indicating that the proteome collection encompassed microenvironments and that the data had a high level of coverage, without substantial bias (**Figure 1C**, **Supplementary Table S3**). Thus, the datasets provided a comprehensive proteome reference map for the aboveground and underground components of foxtail millet seedlings.

### Quantitative proteomics analysis of leaf and root tissues under drought and recovery conditions

To facilitate comparisons between different treatment groups, protein abundances were analyzed with iBAQ, and the iBAQ values of samples were normalized as FOT values (Schwanhäusser et al., 2011). As previously conducted, a stringent threshold of fold change (drought/control-D; recovery/control-R) >1.5 and <0.67 were used as cutoffs to identify statistically significant DAPs. Under drought stress, 773 and 649 DAPs were identified in leaf and root tissues, respectively (**Supplementary Table S4**). A total of 952 (in leaf) and 779 (in root) DAPs were also identified after rewatering (**Supplementary Table S5**). The fold change values of up- and downregulated proteins in leaves and roots during drought stress were primarily within 0.5–2-fold typical values. However, the abundance of upregulated proteins in leaves and roots decreased after rewatering, while the abundances of downregulated proteins significantly increased (**Supplementary Figure S4B**). A total of 658 DAPs were identified in leaves, 534 in roots, and 115 in both tissues during drought stress (**Supplementary Figure S5A**). After rewatering, 730 DAPs were identified in leaves, 557 in roots, and 222 in both roots and leaves (**Supplementary Figure S5A**). The PCA of DAP profiles across all treatments also confirmed that leaves and roots exhibited different proteomic responses to drought and recovery (**Supplementary Figures S5B–D**). Thus, significant differences in the proteomes of leaves and roots were apparent under either drought or recovery conditions in foxtail millet plants.

### Different functional classifications of responsive proteins in leaves and roots during drought and recovery

**Figure 2:**
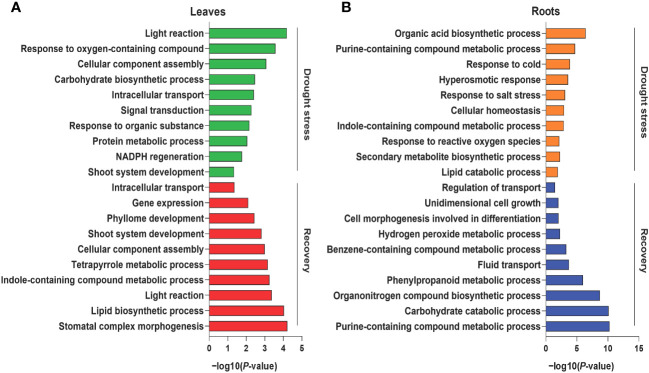
Gene Ontology (GO) enrichment analysis of differentially abundant proteins under drought and recovery conditions. Representative GO terms of the enriched proteins in leaf tissues **(A)** and root tissues **(B)** under drought stress and recovery. The X axis represents the significant degree of pathway enrichment.

Tissue-specific proteins were identified based on the abovementioned categorizations to better understand the biological processes of leaf and root tissues during drought stress and recovery in foxtail millets. During drought stress, 334 and 233 DAPs were specifically enriched in the leaf and root tissues, respectively (**Supplementary Figure S5A**). GO enrichment analysis revealed that the functions of leaf tissue-specific proteins were primarily related to photosynthesis, signal transduction, and metabolic processes (e.g., amino acid carbohydrate metabolism) (**Figure 2A**, **Supplementary Table S6**). Enriched proteins specifically expressed in roots were related to responses to stress, hormone synthesis, secondary metabolite biosynthesis, and homeostasis (**Figure 2B**, **Supplementary Table S6**). A total of 423 DAPs were specifically enriched in leaf tissues after rewatering, and their functions were primarily related to development and light reactions (**Figure 2A**, **Supplementary Figure S5A**, **Supplementary Table S6**). In addition, the functions of 294 DAPs expressed in roots during recovery were related to secondary compound metabolism and development (**Figure 2B**, **Supplementary Figure S5A**, **Supplementary Table S6**).

**Figure 3:**
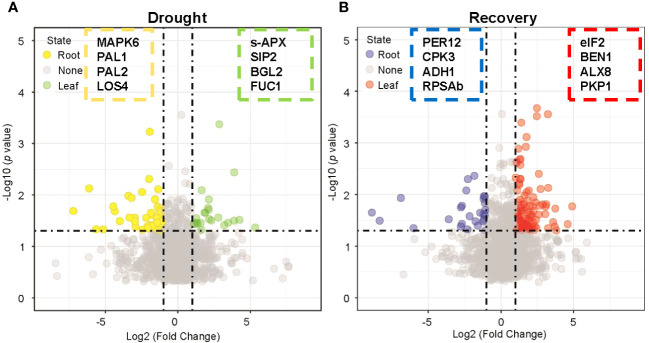
Nominating potential drought-resistant proteins in leaf and root tissues. **(A)** Volcano plot displaying the differentially expressed proteins in leaf and root tissues under drought stress by applying a twofold change expression difference with *p* < 0.05 (Student’s *t*-test). Proteins significantly enriched in leaf/root tissues were highlighted with green/yellow-filled circles. MAPK6: *Seita.4G069900*, PAL1: *Seita.6G181000*, PAL2: *Seita.7G168700*, LOS4: *Seita.9G534300*, s-APX: *Seita.7G102200*, SIP2: *Seita.7G139800*, BGL2: *Seita.3G315000*, FUC1: *Seita.7G197500*. **(B)** Volcano plot displaying the differentially expressed proteins in leaf and root tissues under drought recovery condition by applying a twofold change expression difference with *p* < 0.05 (Student’s *t*-test). Proteins significantly enriched in leaf/root tissues were highlighted with red/blue-filled circles. PER12: *Seita.3G004800*, CPK3: *Seita.5G231500*, ADH1: *Seita.8G099100*, RPSAb: *Seita.9G517100*, eiF2: *Seita.3G071100*, BEN1: *Seita.2G354300*, ALX8: *Seita.7G280700*, PKP1: *Seita.9G055900*.

Tissue-specific DAPs with twofold abundance changes that were statistically significant (*p* < 0.05, Student’s *t*-test) were also identified during drought and recovery. Drought stress enhanced the abundance of 23 and 44 proteins that satisfied the abovementioned criteria in leaves and roots, respectively (**Figure 3A**, **Supplementary Table S7**). Protein abundance was altered to a greater extent in roots than in leaves during drought stress. In contrast, rewatering caused more obvious changes of protein abundance in leaves than in roots. These results reflect that proteins from leaves are primarily involved in metabolic processes, while proteins in roots are primarily involved in maintaining the stability of root cell environments and regulating responses to drought. When water becomes available again, the abundance of proteins in leaves is primarily associated with photosynthesis and leaf development. In contrast, protein abundance in roots is primarily involved in regulating secondary metabolite metabolism and root cell development. The results overall indicated that the proteomes of leaves differed from those in roots in response to drought and subsequent recovery.

### Transcriptional analysis of DAPs

**Figure 4:**
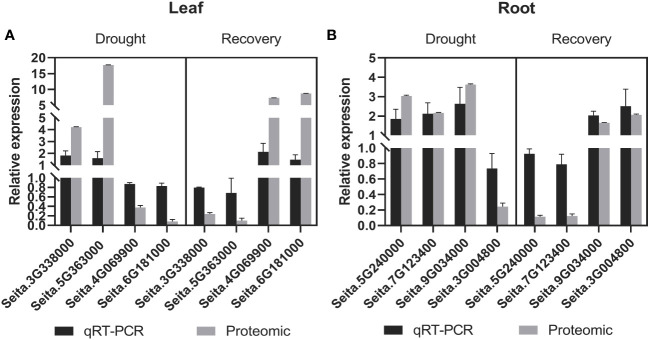
Comparative analysis of the mRNA and protein levels of the selected genes and their corresponding proteins. **(A)** In leaf tissues, a comparative analysis of the candidate proteins in mRNA and protein levels under drought and recovery conditions. **(B)** In root tissues, a comparative analysis of the candidate proteins in mRNA and protein levels under drought and recovery conditions. The expression levels were calculated by the 2^-ΔΔCT^ method. Three biological replicates for each gene were performed, and the values of gene expression are shown as mean ± SD. Protein values are means ± SD (*n* = 4).

The RNA expression of eight randomly selected genes encoding candidate drought-responsive proteins from leaves and roots during drought stress and recovery that were identified with comparative proteomics was validated using quantitative RT-PCR (qRT-PCR) analysis. Specifically, four genes from leaves were evaluated, including those encoding the lipid transport superfamily protein, mitogen-activated protein kinase 6 (MPK6), and non-specific lipid-transfer protein, phenylalanine/tyrosine ammonia-lyase (PAL) (i.e., genes Seita.3G338000, Seita.4G069900, Seita.5G363000, and Seita.6G181000) (**Figure 4A**). Four genes from roots (Seita.3G004800, Seita.5G240000, Seita.7G123400, and Seita.9G034000) that encode peroxidase (PER12), carbonic anhydrase (CA2), glyceraldehyde-3-phosphate dehydrogenase (GAPA-2), and actin depolymerizing factor (ADF11), respectively, were also investigated (**Figure 4B**). The expression of genes with upregulated protein levels was also induced at the transcriptional level during drought stress and recovery conditions. In contrast, proteins with downregulated abundance did not exhibit any significant changes at the transcriptional levels. These differences might be due to differences in post-transcriptional and post-translational regulation.

### Tissue-specific proteins are associated with functional changes during drought and recovery

**Figure 5:**
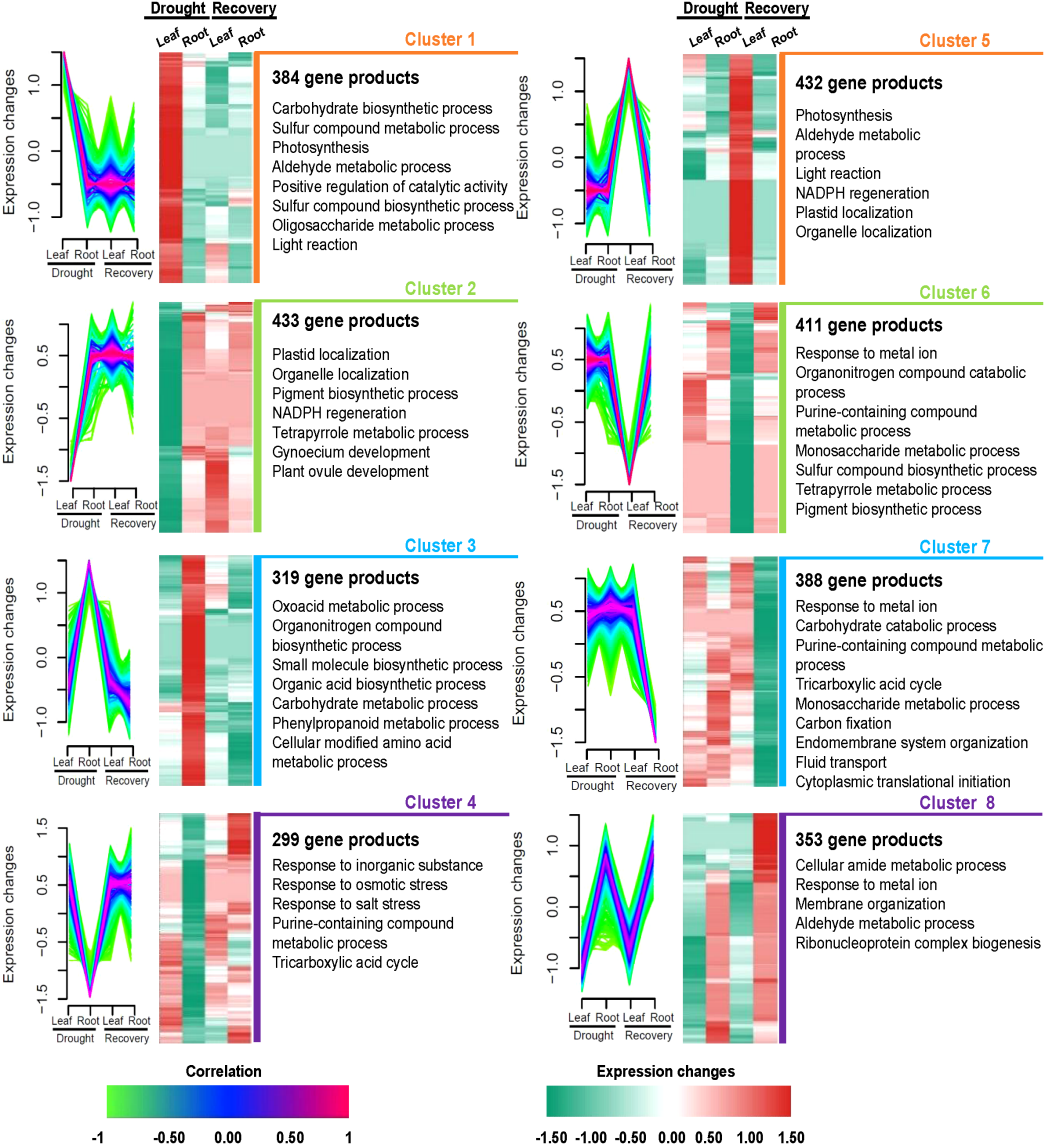
Tissue-specific protein clusters and functional differences under drought and recovery conditions in leaves and roots. Eight protein clusters were revealed by co-expression analysis. Left panel: the co-expression patterns of the proteins in each cluster; right panel: representative Gene Ontology terms of each cluster.

The co-expression analysis of 3,019 highly abundant proteins (absolute |log_2_FC| >0 in at least one treatment in leaf or root samples; **Supplementary Table S8**) yielded eight protein clusters that might correspond to the independent functions of Yugu1 leaves and roots during responses to drought and recovery. The abundance levels of 384 proteins in leaves that comprised cluster 1 were significantly higher than in roots under drought stress. Notably, these 384 proteins were also expressed at low levels in leaves and roots when treated with rewatering and as compared with leaves in the drought treatment. Cluster 2 comprised 433 gene products with relatively low abundance levels during drought stress of leaves compared with drought stress of roots and the recovery treatments of leaves and roots. Clusters 1 and 2 exhibited differences in protein abundance during drought stress of leaves that were apparently associated with leaf responses to drought, including *via* photosynthesis, plastid localization, positive regulation of catalytic activity, and NADPH regeneration (**Figure 5**, **Supplementary Tables S8**, **S9**). Cluster 3 comprised 319 relatively upregulated proteins under drought stress in roots. In contrast, cluster 4 comprised 299 relatively downregulated proteins. Clusters 3 and 4 were associated with responses of roots to drought and were annotated as having functions related to carbohydrate metabolism, phenylpropanoid metabolism, and responses to osmotic stress (**Figure 5**, **Supplementary Tables S8**, **S9**).

During the recovery treatment of leaves, 432 proteins were significantly upregulated in cluster 5, while 411 gene products were significantly downregulated and comprised cluster 6. Clusters 5 and 6 were characterized by roles in photosynthesis, porphyrin-containing compound metabolism, and protein catabolism while also being associated with DAPs in leaves after rewatering (**Figure 5**, **Supplementary Tables S8**, **S9**). A total of 388 proteins were classified into cluster 7 that were all downregulated only in roots after rewatering. Interestingly, the 353 proteins comprising cluster 8 were upregulated during drought and rewatering in roots. Biological processes were primarily associated with cellular amide metabolism, responses to metal ions, membrane organization, aldehyde metabolism, and ribonucleoprotein complex biogenesis (**Figure 5**, **Supplementary Tables S8**, **S9**).

## Discussion

Drought causes declines in cereal production and is frequently occurring in regions due to increasing extreme global climate events. Foxtail millet is one of the most widely grown millet crops, especially in the arid and semi-arid regions of Asia, North Africa, and the Americas and is an ideal model plant for researching drought resistance (Lata et al., 2013; Yang et al., 2020). Increasing numbers of studies have revealed the molecular mechanisms of drought resistance in foxtail millet using transcriptomic and proteomic approaches. Nevertheless, a more comprehensive understanding of the mechanisms underlying crop physiological responses to drought stress is needed to contextualize variation in the proteomes of leaves and roots among distinct temporal and spatiotemporal dimensions during drought stress and recovery. In this study, comparative proteomics was used to generate insights into the proteomes of foxtail millet leaves and roots during drought stress and recovery conditions, in addition to understanding the altered signaling pathways of leaves and roots during drought stress and recovery at the proteomic level.

### The physiology and morphology of foxtail millet responses to drought stress and rewatering

Crops are sessile organisms that comprise aboveground and underground morphological components that exhibit entirely different behaviors in response to drought stress. When plants experience drought stress, the aboveground components will initiate responses to mitigate drought effects, like partial or entire stomatal leaf closure, resulting in reduced photosynthesis, inhibited element and nutrient transport, and leaf wilting, such that sustained drought stress significantly affects crop yields (Yang et al., 2005; Schwanhäusser et al., 2011; Choudhury et al., 2022). In addition, root systems comprise organs that directly absorb water from the soil and will invade deeper in soils to achieve absorption of more water. However, root coefficients, densities, and weights are significantly reduced after severe drought stress (Shinozaki et al., 2003; Yang et al., 2005; Farooq et al., 2009; Kantar et al., 2011). The RWC is an indicator of plant water status and can reflect the level of drought stress in plants. The drought stress level can be categorized into three levels by RWC: mild (water loss 8%–10%), moderate (water loss 10%–20%), and severe stress (water loss of more than 20%) (Hsiao, 1973). In this study, foxtail millet leaves were wilted under drought stress to evaluate physiological changes relative to normal cultivation conditions. Lower RWCs (water loss of 8.4%) and chlorophyll concentrations were observed in drought-stressed plants in addition to increased REL (**Supplementary Figure S1**). Thus, mild drought altered the physiological states of aboveground components (leaves) of the plants and inhibited their normal growth. When water was replenished to allow recovery, the indicators of the aboveground components gradually returned to normal levels, and no differences were observed relative to the control groups (**Supplementary Figure S1**). The aboveground and underground components of plants nevertheless operate as a single organism during drought stress and reasonably allocate resources to regulate individual growth and development (Magnani et al., 2000). Unlike the aboveground components, root lengths and surface areas increased during drought stress (**Supplementary Figure S2**). Specifically, significant differences were not observed between the control and recovery groups after rewatering (**Supplementary Figure S2**). Thus, when foxtail millets perceive a lack of water, the growth of the root systems accordingly change to ensure that they can absorb moisture from deeper soils and slow or avoid damage caused by drought to maintain the growth and development of aboveground organs.

### Significant differences in the functions of proteins enriched in roots and leaves under drought stress

Comparative proteomics analysis was used to identify the protein profiles of leaves and roots in response to drought stress. The PCA revealed that the proteins of leaf and root tissues responsive to drought were highly distinct (**Supplementary Figures S5B–D**). This implied that the functions of proteins in leaves and roots might also differ in response to drought stress. Consistently, previous reports have shown that plant leaves and roots exhibit significantly different responses to drought stress (Hui et al., 2015; Liu et al., 2015; Zhang F et al., 2017). In this study, some proteins were highly expressed in leaf tissues during drought stress (**Figure 3A**). The co-expression analysis revealed that 384 and 433 proteins were significantly up- and downregulated in leaves, respectively (**Figure 5**, **Supplementary Tables S8**, **S9**). Homologs of proteins highly expressed in leaves during drought stress have previously been shown to be associated with stress tolerance—for example, proteomic data revealed that the abundance of Seita.7G102200 (stromal ascorbate peroxidase) was significantly upregulated in leaf tissues compared to root tissues during drought stress. Stromal ascorbate peroxidase is critical for removing H_2_O_2_ in plants and is found in chloroplasts, where it has been shown to play critical roles in responding to abiotic stress in *Arabidopsis thaliana* (Heiber et al., 2014; Li et al., 2019). AtSIP2 is an alkaline alpha-galactosidase with substrate specificity for raffinose and is strongly expressed in sink leaves—sink organs for assimilate are net importers of assimilate—where it metabolizes raffinose from sink tissue (Peters et al., 2010). The abundance of Seita.7G139800 (a homolog of *AtSIP2*) was upregulated in leaves rather than in roots during drought stress. *AtBGL2* is involved in drought stress and leaf senescence in *A. thaliana* (Liu et al., 2013; Borniego et al., 2020). In this study, Seita.3G315000 (a homolog of AtBGL2) exhibited more variable abundance in leaf tissues compared to roots during drought stress. AtFUC1 encodes alpha-L-fucosidase and was homologous to Seita.7G197500. A previous study revealed that the expression of alpha-L-fucosidase was highly upregulated in the leaves of hot peppers under drought stress (Nallamothu et al., 2020). The protein abundance of Seita.7G197500 (FUC1) was likewise higher in the leaves of our study than in the roots during drought stress.

Some proteins were expressed at higher levels in roots than in leaves after drought stress (**Figures 3A**, **5**; **Supplementary Tables S8**, **S9**), with some of these having homologous proteins previously observed in foxtail millet root and whose functions have previously been reported, including MAPK6, CA2, PAL1/2, and LOS4. Seita.4G069900, which was predicted to encode MAPK6 and MAPK6 (AT2G43790), is primarily expressed in the apical regions of root meristems in *A. thaliana* and in the root transition zone, where it has been implicated in regulating cell division and root growth (Müller et al., 2010). Furthermore, previous studies have shown that several components of the MAPK cascade are involved in ABA signaling and responses to drought stress (Yang et al., 2010; Li et al., 2016; Yang & Guo, 2018; Chen et al., 2021). Phenylalanine ammonia-lyase (PAL, AT2G37040) is required for normal plant growth, development, and adaptation to various environmental stresses (Huang et al., 2010). *PtrWRKY75* regulates the expression of *PAL1/2* in poplar trees to improve water use efficiency during drought stress, with *PAL2* particularly expressed in the roots (Gray-Mitsumune et al., 1999; Zhang et al., 2020). Here the abundance of Seita.6G181000 (PAL1) and Seita.7G168700 (PAL2) was dramatically increased in roots compared to leaves under drought stress. Furthermore, Seita.9G534300 abundance was significantly upregulated in roots during drought stress, with the gene homolog *LOS4* (AT3G53110) known to encode a DEAD-Box RNA helicase. LOS4 may also be involved in plant stress responses *via* the negative regulation of *DREB* expression (Gong et al., 2002; Gong et al., 2005). The results from this study are consistent with these previous observations. Greater protein changes were observed in leaves during drought compared to roots. These observed changes in protein abundance levels under drought stress conditions suggest a possible role in stress tolerance. Overall, these results demonstrate that the functions of drought-induced proteins significantly differed among leaves and roots. Thus, different pathways may also respond to drought in leaves and roots.

### Functional variation of enriched proteins in roots and leaves under rewatering conditions

Recovery treatment after drought stress increased the abundance of plant growth-related proteins and decreased the abundance of numerous drought-responsive proteins (Hao et al., 2015). DAPs between leaves and roots after rewatering were investigated in detail (**Figures 3B**, **5**; **Supplementary Tables S8**, **9**). After rewatering, the abundance levels of Seita.5G231500 (CPK3) and Seita.1G032400 (CPK4) were substantially higher in leaves than in roots. AtCPK3 and AtCLPS3 of *Arabidopsis* have previously been shown to be associated with plant growth. Specifically, calcium-dependent protein kinase 3 (CPK3) has been observed as expressed in guard cells and mesophyll cells, where it functions to regulate guard cell ion channels and plant growth in *A. thaliana* (Mori et al., 2006). In addition, the nuclear protein (CLPS3) acts on mRNA processing and is involved in phyllome development in *A. thaliana* (Xing et al., 2008). Moreover, several proteins upregulated after rewatering were similar to stress-related marker proteins in *A. thaliana*, including NF-YB3 (AT4G14540, homolog of Seita.9G36570) (Kumimoto et al., 2008; Feng et al., 2015), SNRK2.4 (AT1G10940, homolog of Seita.7G100500) (Song et al., 2022), and MAPK6 (AT2G43790, homolog of Seita.4G069900) (Xin et al., 2021). In this study, the protein abundance levels of Seita.9G365700 (NF-YB3) and Seita.7G100500 (SNRK4) were significantly downregulated in leaves after rewatering. This result is consistent with those of others, wherein the abundance of some drought-related proteins decreased after recovery (Hao et al., 2015). Interestingly, we observed that Seita.4G069900 (MAPK6) was upregulated in roots after drought stress and upregulated in leaves after recovery. In contrast, Seita.7G197500 (FUC1) abundance was upregulated in leaves during drought stress and in roots following recovery. These results suggest that the functions of this protein differed in leaves and roots in response to drought stress and recovery. It is worth noting that the number of significantly altered proteins in roots after rewatering was markedly higher than during drought stress (**Figure 5**, **Supplementary Tables S8**, **S9**). These results suggested that greater numbers of proteins were mobilized in roots during rewatering after drought stress. Overall, the significant differences in leaf and root responses to recovery might indicate differences in the specific pathways involved in recovery within leaf and root tissues. Furthermore, these insights help form a better understanding of how protein abundance is altered in leaves and roots during drought stress recovery.

### Overview of metabolic pathways involved in foxtail millet response to drought stress and recovery

**Figure 6:**
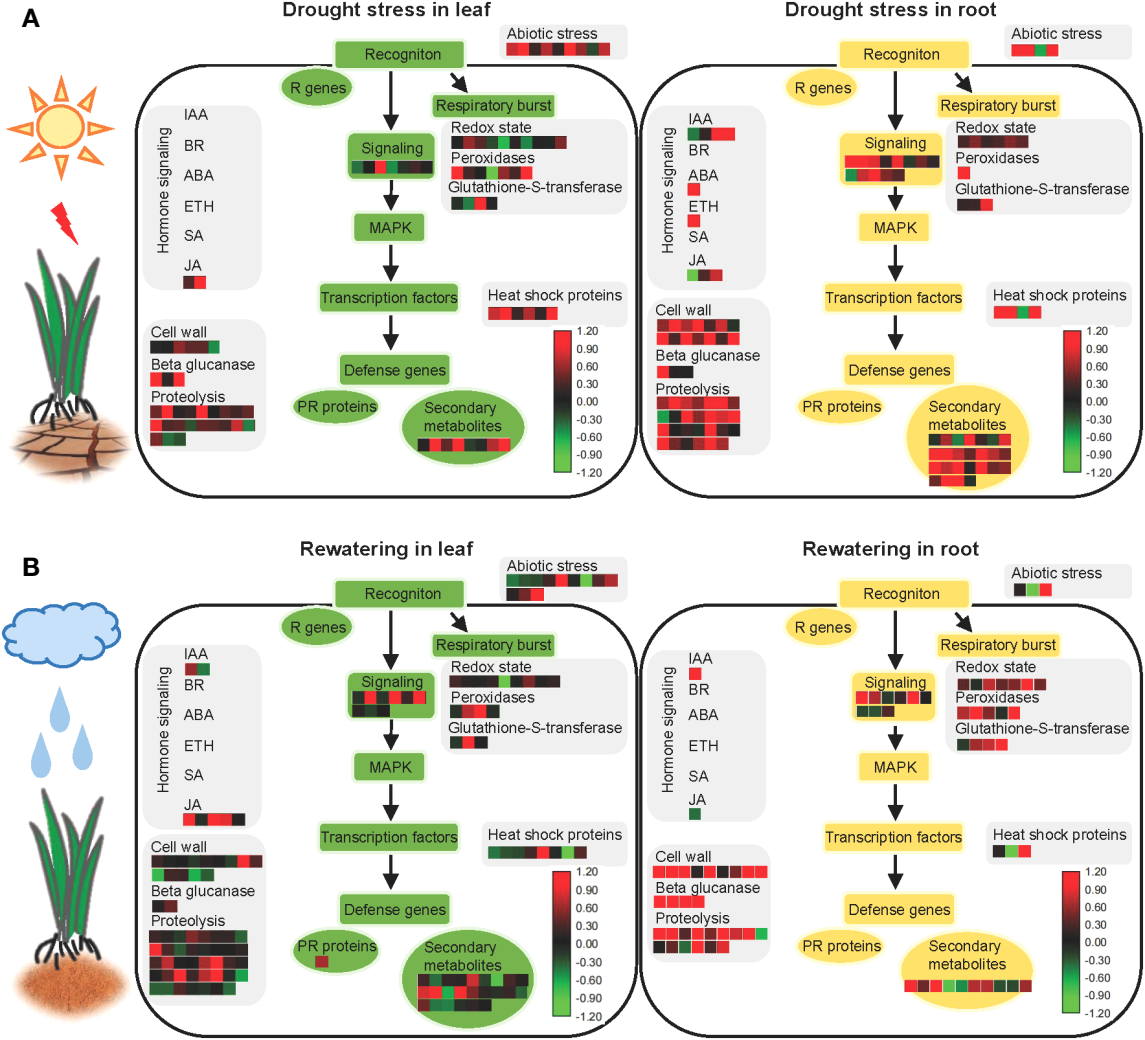
Model showing the responses of aboveground and underground parts of foxtail millet (*Setaria italica* L.) to drought stress and rewatering. **(A)** MAPMAN overview of drought stress pathways in aboveground (leaf) and underground (root) parts of foxtail millet (*Setaria italica* L.). **(B)** MAPMAN overview of rewatering pathways in aboveground (leaf) and underground (root) parts of foxtail millet (*Setaria italica* L.). Each colored cell represents the value of the normalized log_2_ fold change according to the color scale at the bottom of the figure. Red: increased levels; green: decreased levels: black: no changes.

All proteins identified in the roots and leaves during drought stress and rewatering were used to create model maps that depicted their overall involvement in stress responses (**Figure 6**, **Supplementary Table S10**). Significant differences of abundance between leaves and roots were primarily observed among secondary metabolic pathways in hormone regulation and cell wall synthesis during drought stress and rewatering conditions.

After sensing environmental stresses, plants start to accumulate secondary metabolites that can improve their viability (Isah, 2019). Drought is a frequently occurring environmental stress that significantly impacts secondary metabolite pathways. Indeed many studies of various crops have shown increased levels of endogenous secondary metabolites in response to drought stress—for example, genes related to flavonoid biosynthesis and lignin biosynthesis pathways were upregulated during drought stress of cotton, leading to polyphenol and lutein biosynthesis (Yildiz-Aktas et al., 2009; Ranjan et al., 2012a). Furthermore, lignin, terpenoids, and terpenes accumulated in plants during drought stress (Jogawat et al., 2021). Moreover, drought can induce the production of free amino acids that primarily act as osmoprotectants and antioxidants during drought stress (Vranova et al., 2010; Jogawat et al., 2021).

As a precursor response to external signals, roots promote the rapid accumulation of secondary metabolites at first that makes it possible to adapt to water-limited conditions and maintain homeostasis—for example, cyanogenic glucoside contents in cassava roots were enhanced during drought stress (Imakumbili et al., 2019). Lignin also accumulated in chickpea roots during drought due to the downregulation of the *LACCASE2* gene by *miR397b* because the *LACCASE* family enzymes negatively regulate lignin accumulation (Sharma et al., 2020). Furthermore, the ectopic expression of *CKX* in barley plants results in stronger lignification of root tissues and activates the biosynthesis of flavonoids and antioxidants (Nakabayashi et al., 2014; Vojta et al., 2016; Jogawat et al., 2021). In addition, drought-induced terpenoid phytoalexins are root-specific in maize but do not affect the phytoalexin levels in aboveground components (Vaughan et al., 2015). Accumulated secondary metabolites in roots were also observed in this study, with 25 differently expressed proteins enriched in roots under drought stress but only eight differently expressed proteins enriched in leaves (**Figure 6**, **Supplementary Table S10**)—for example, PAL2 is involved in secondary metabolism and was highly expressed in roots (**Supplementary Table S10**). Interestingly, the number of differently expressed proteins involved in secondary metabolic pathways that were enriched in roots decreased during recovery, but increased in recovery of leaves, contrasting with results from the drought stress groups. Overall, these results provide new evidence for secondary metabolite responses of plants following drought stress and recovery.

Hormones play important roles in response to environmental stress, and water stress directly affects the hormonal concentrations in plants. ABA hormones play important roles in regulating plant responses to drought stress (Finkelstein, 2013). Specifically, ABA signaling effectively regulates stomatal activity, ROS stabilization, and secondary metabolite synthesis under drought stress (Hubbard et al., 2010). Overall, roots sense water deficit signals in soils and subsequently transmit the signal to the aboveground components of plants during drought stress, eventually leading to a response such as closing of the leaf stomata. Some studies have shown that ABA is synthesized in roots during drought stress, followed by rapid translocation of ABA throughout plants (Christmann et al., 2004; Endo et al., 2008). During drought stress, CLE25 peptides are expressed in root vascular tissues and move through the vascular system to the leaves, where they induce NCED3 expression and enhance ABA accumulation therein, thereby inducing stomatal closure and improving overall plant water balance (Takahashi et al., 2018; Gupta et al., 2020). In this study, NCED4 was observed in foxtail millet as a homolog of NCED3 in *A. thaliana* (**Supplementary Table S10**). Furthermore, when the plants experienced drought stress, more responsive proteins involved in hormone signaling were identified in roots than in leaves (**Figure 6**, **Supplementary Table S10**).

An analysis of stress pathways revealed that more proteins related to cell wall synthesis were identified in roots, but significantly fewer proteins involved in cell wall synthesis pathways were upregulated in leaves. This could be related to morphological changes in root structures during water deficits, thereby requiring cell wall synthesis and remodeling to complete cell division and cell elongation (Gupta et al., 2020), hence the enhancement of water and nutrient absorption (Tenhaken, 2015; Rellán-Álvarez et al., 2016; Dinneny, 2019). Osmotic stress is the primary signal of drought (Zhu, 2016). Osmotic stress can induce the production of oxidase that crosslinks structural proteins in cell walls and eventually hardens cell walls (Csiszár et al., 2012; Ranjan et al., 2012b). During prolonged osmotic stress, high levels of ROS are produced from hydroxyl radicals (OH^•^) that cleave the glycan bonds in plant polysaccharides (Fry, 1998; Schopfer, 2002; Renew et al., 2005). Furthermore, swelling proteins and xyloglucan-modifying enzymes are highly expressed under osmotic stress, which loosen cell walls to ensure normal plant development (Rose et al., 2002; Cho et al., 2006; Tenhaken, 2015). Consistent with the results of this study, cell wall synthesis-related proteins have been shown to play an important role in the drought resistance of foxtail millet.

During drought stress, plants do not maintain a balance between water loss and absorption. Consequently, different drought mechanisms are used to avoid or tolerate the dehydration of leaves and roots (Zhu, 2002). However, a significant gap remains in our understanding of the molecular mechanisms used by the leaves and roots of plants during drought stress and rewatering conditions. Consequently, the analysis of proteomes of the leaves and roots of foxtail millet provides new insights to help better understand how they respond to drought stress and rewatering.

There are some limitations of this study. First, a universally accepted foxtail millet protein database is currently not available for the identification and functional annotation of proteins. Furthermore, the potential for omission of tissue-specific proteins cannot be excluded, although these would be unlikely to alter the overall conclusions of the study. Second, although these data improve our understanding of the responses of leaves and roots to drought stress and rewatering in foxtail millet, the involvement and fine-scale regulation of such pathways requires further investigation *via* the development and characterization of designed mutants.

## Conclusion

Foxtail millet (*Setaria italica* L.) has long been used as a model to understand stress responses in plants. In this study, physiological analysis demonstrated that drought stress reduced the RWC and chlorophyll contents of leaves, but increased the REL, while the lengths and surface areas of root systems increased. Moreover, the physiological status of leaves and root systems after rewatering was similar to the controls. The comparative proteomic analysis confirmed that the proteins in the leaves and roots of foxtail millets differed under drought stress and rewatering conditions. Proteins from leaves in response to drought conditions were primarily involved in metabolic processes. In contrast, proteins in roots under these conditions were primarily associated with maintaining the stability of root cell environments, consistent with observed physiological phenotypes. After rewatering, proteins identified in leaves were primarily associated with photosynthesis and leaf development. In contrast, responsive proteins in roots were primarily associated with the metabolic regulation of secondary metabolites and root cell development. Highly co-expressed proteins in leaves and roots in drought and rewatering were clustered into eight groups based on their functions. Concomitantly, apparent discrepancies in the regulatory signaling pathways between leaves and roots in response to drought stress and rewatering conditions primarily involved secondary metabolite pathways, hormone regulation, and cell wall synthesis. Overall, proteomic analysis provided extensive insights into the mechanisms of drought tolerance and recovery in the leaves and roots of foxtail millet.

## Data availability statement

The datasets presented in this study can be found in online repositories. The data presented in the study are deposited in the iProX repository, accession number IPX0003683000.

## Author contributions

HG, WG, HZ, and LB contributed to the conception and design of the study. TZ and LZ organized the database. JL and JS performed the statistical analysis. HG and HZ wrote the first draft of the manuscript. WG, HZ, LB, and WG wrote sections of the manuscript. All authors contributed to the article and approved the submitted version.
