# Supplementary material for: Proteomic analysis of leaves and roots during drought stress and recovery in Setaria italica L
Source: Front Plant Sci. 2023 Oct 11;14:1240164. doi: 10.3389/fpls.2023.1240164 (PMC10598781; doi:10.3389/fpls.2023.1240164)
Supplement: Supplementary file 1 [file DataSheet_1.pdf]

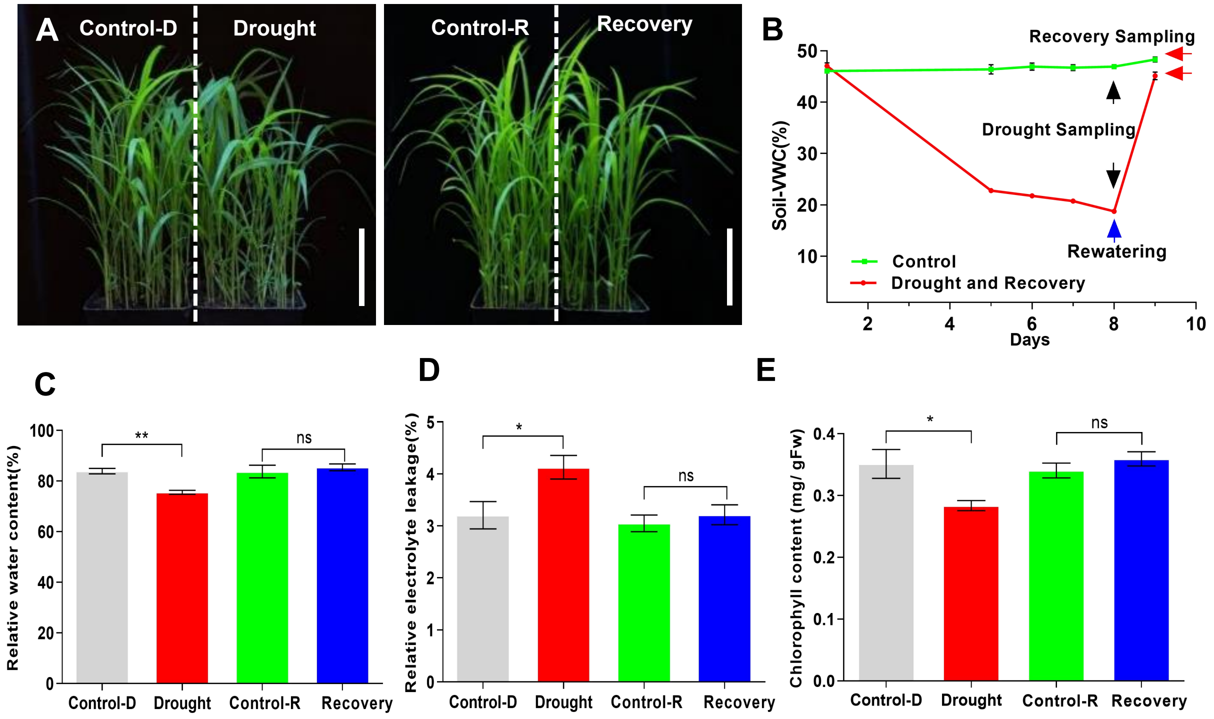

Figure S1. Morphological and physiological analysis of the effects of drought stress and drought-recovery on foxtail millet (*Setaria italica* L.). (A) Phenotypes of Yu1 seedlings under normal condition (Control-D and Control-R), 8 days after drought stress (Drought) and 8 days after drought stress with recovery for one day (Recovery). Bar = 5 cm. (B) Soil volumetric water content during the 8-day drought treatment and recovery for one day. The arrow indicates the sampling date for differential proteomic analysis. Data were collected from four independent experiments. Comparison of the relative water content (C), the relative electrolyte leakage (D) and the chlorophyll content (E) under normal condition (Control-D) and 8 days after drought stress (Drought), normal condition (Control-R) and with recovery for one day (Recovery). For B, C, D and E, error bars showed SE and the asterisks indicated the significant differences from the control (*, $P < 0.05$ and **, $P < 0.01$, calculated using One-Way ANOVA). Data were collected from four independent experiments, and more than 30 plants were collected each time (n > 90).

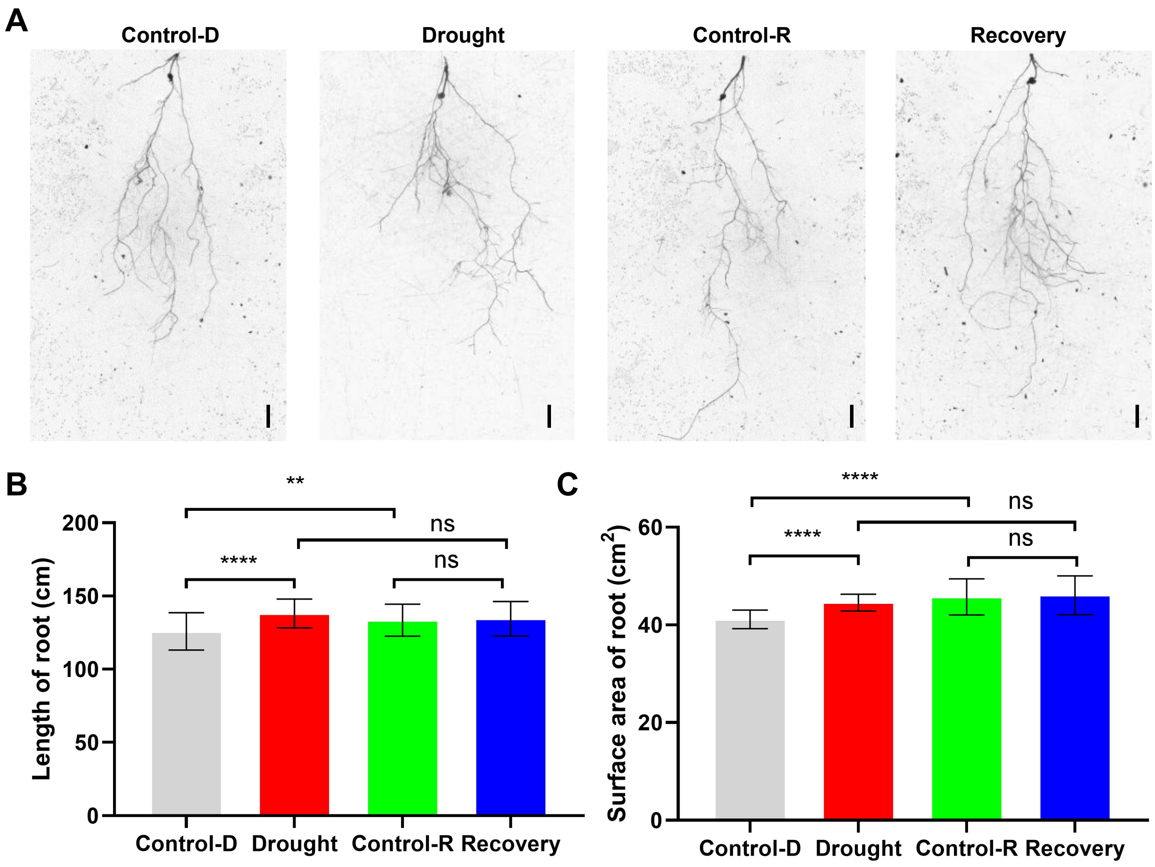

Figure S2. Morphological analysis of the effects of drought stress and drought-recovery on the root of foxtail millet (*Setaria italica* L.). (A) The morphology of Yu1 roots under normal condition (Control-D and Control-R), 8 days after drought stress (Drought) and 8 days after drought stress with recovery for one day (Recovery). Bar = 5 mm. (B) Comparison of the total root length under normal condition (Control-D) and 8 days after drought stress (Drought), normal condition (Control-R) and recovery for one day (Recovery). (C) Comparison of the surface area of roots under normal condition (Control-D) and 8 days after drought stress (Drought), normal condition (Control-R) and drought-recovery for one day (Recovery). For B and C, error bars showed SE and the asterisks indicated the significant differences from the control (**, $P < 0.05$； ****, $P < 0.0001$ calculated using two tail T-test). Data were collected from two independent experiments, and more than 15 plants were collected each time ($n \geq 30$).

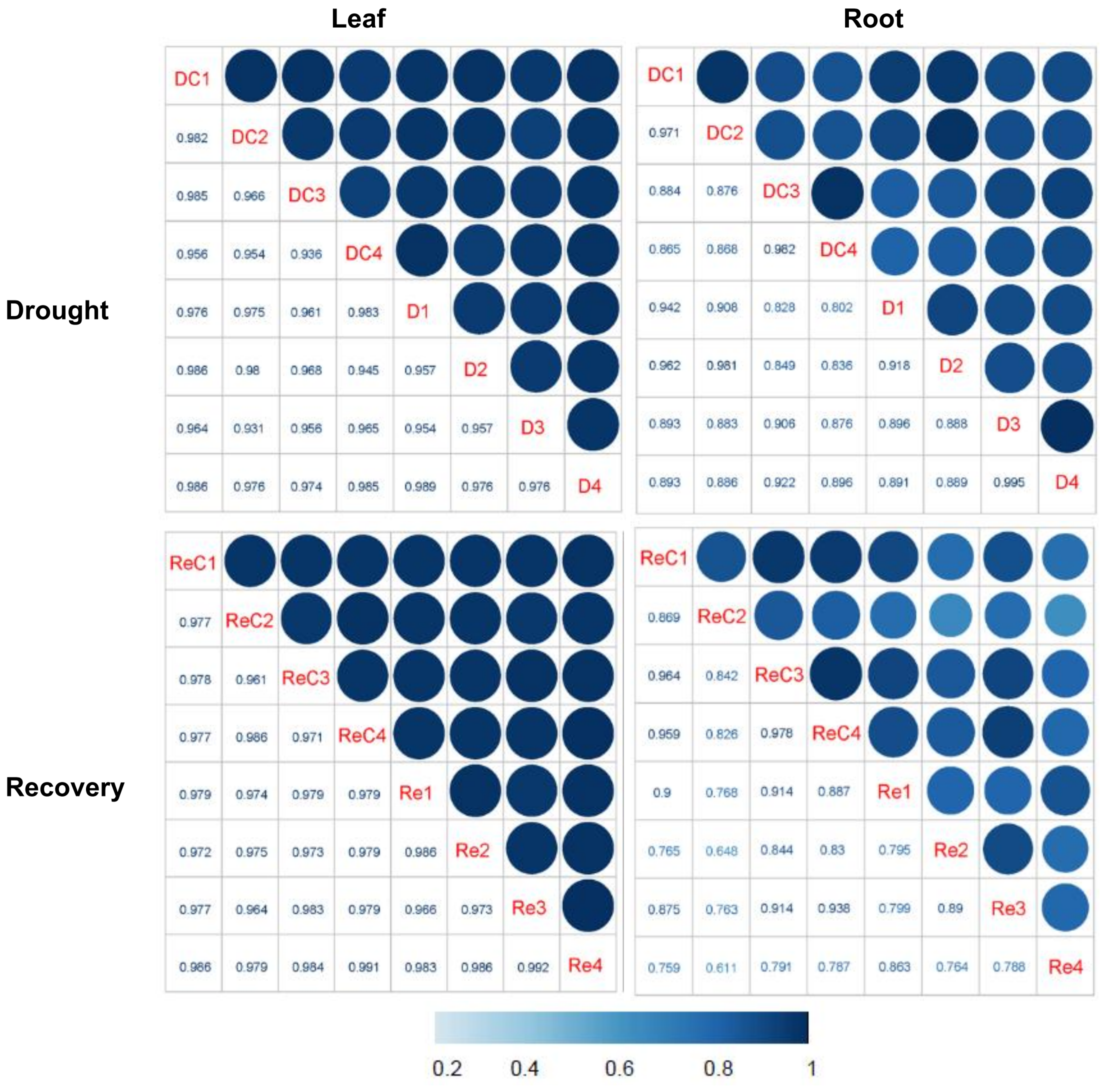

Figure S3. The correlation matrix among the proteomic datasets of four biological repetitions in leaf and root tissues: DC (Control-D), D (Drought), ReC(Control-R), Re(Recovery). The depth of numbers and colors represents the correlation coefficient.

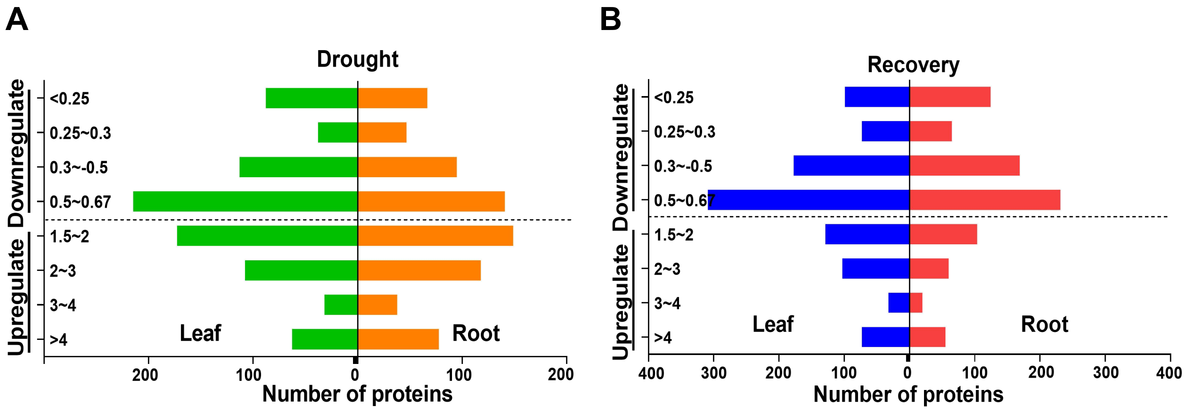

Figure S4. The distribution of differentially expressed proteins in leaf and root. (A) The distribution of differentially expressed proteins under drought stress compared with the control in leaf and root. Y axis showed the fold changes of protein abundance under drought stress compared with the control (cutoff of over 1.5 for increased expression and less than 1/1.5 (0.67) for decreased expression). (B) The distribution of differentially expressed proteins under drought-recovery condition compared with the control in leaf and root. Y axis showed the fold changes of protein abundance under drought-recovery compared with the control (cutoff of over 1.5 for increased expression and less than 1/1.5 (0.67) for decreased expression).

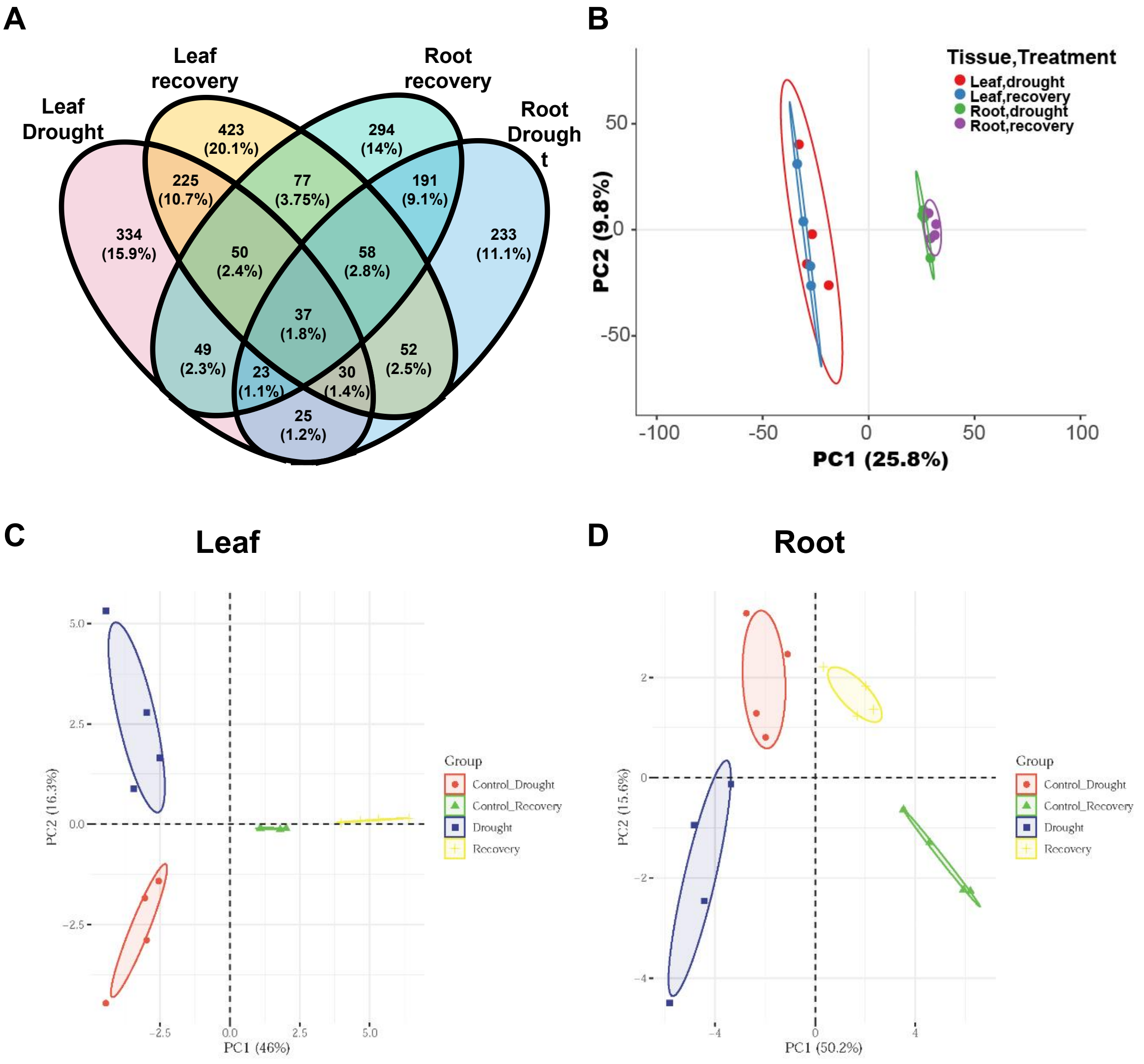

Figure S5. Quantitative analysis of differentially abundant proteins (DAPs) of drought-responsive and recovery-responsive proteins in leaf and root tissues of foxtail millet (*Setaria italica* L.). (A) Venn and upset diagram analyses of the differentially expressed proteins. The number of differentially expressed proteins under drought stress and recovery were shown in the different segments. Principal component analysis (PCA) of the 16 samples of different tissues (B), leaf (C) and root (D) tissue contained four biological replicates under drought and recovery treatment. The percentage is the contribution of this principal component to the variance of the population.
